# PROX1 is a transcriptional regulator of MMP14

**DOI:** 10.1038/s41598-018-27739-w

**Published:** 2018-06-22

**Authors:** Silvia Gramolelli, Jianpin Cheng, Ines Martinez-Corral, Markus Vähä-Koskela, Endrit Elbasani, Elisa Kaivanto, Ville Rantanen, Krista Tuohinto, Sampsa Hautaniemi, Mark Bower, Caj Haglund, Kari Alitalo, Taija Mäkinen, Tatiana V. Petrova, Kaisa Lehti, Päivi M. Ojala

**Affiliations:** 1 Research Programs Unit, Translational Cancer Biology, University of Helsinki, Helsinki, Finland; 2 Department of Oncology, CHUV and University of Lausanne, Switzerland and Ludwig Institute for Cancer Research, Lausanne, Switzerland; 3 Department of Immunology, Genetics and Pathology, Rüdbeck Laboratory, Uppsala University, Uppsala, Sweden; 4 Research Programs Unit, Genome-Scale Biology, University of Helsinki, Helsinki, Finland; 5 Chelsea and Westminster Hospital and Imperial College London, London, UK; 6 Department of Surgery, University of Helsinki and Helsinki University Hospital, Helsinki, Finland; 7 Department of Pathology, University of Helsinki and Helsinki University Hospital, Helsinki, Finland; 8 Department of Microbiology, Tumor and Cell Biology (MTC), Karolinska Institutet, Stockholm, Sweden; 9 Section of Virology, Division of Infectious Diseases, Department of Medicine, Imperial College London, London, UK; 10 Foundation for the Finnish Cancer Institute, Helsinki, Finland

## Abstract

The transcription factor PROX1 is essential for development and cell fate specification. Its function in cancer is context-dependent since PROX1 has been shown to play both oncogenic and tumour suppressive roles. Here, we show that PROX1 suppresses the transcription of *MMP14*, a metalloprotease involved in angiogenesis and cancer invasion, by binding and suppressing the activity of *MMP14* promoter. *Prox1* deletion in murine dermal lymphatic vessels *in vivo* and in human LECs increased MMP14 expression. In a hepatocellular carcinoma cell line expressing high endogenous levels of PROX1, its silencing increased both MMP14 expression and MMP14-dependent invasion in 3D. Moreover, PROX1 ectopic expression reduced the MMP14-dependent 3D invasiveness of breast cancer cells and angiogenic sprouting of blood endothelial cells in conjunction with MMP14 suppression. Our study uncovers a new transcriptional regulatory mechanism of cancer cell invasion and endothelial cell specification.

## Introduction

The transcription factor PROX1 is involved in the development of the central nervous system, lens, heart, liver and pancreas^1–6^. PROX1 is also necessary and sufficient for the differentiation of lymphatic endothelial cells (LECs)^7,8^. The role of PROX1 in cancer is context and tumour type-dependent since it has been shown to have both oncogenic and tumour-suppressive properties^9^. In agreement with the concept that during oncogenesis an aberrant developmental program is activated, altered PROX1 expression is often found in malignant cells of organs, whose normal development depends on PROX1^9^. Glioma, esophageal carcinoma and colon cancer display high PROX1 levels^10–13^ indicative of an oncogenic role, while in hepatocellular carcinoma (HCC) PROX1 expression is reduced, suggesting a tumour-suppressive role^14–16^. Moreover, high expression of PROX1 was recently reported to associate to better survival in gastric cancer^17^.

PROX1 expression was also recently investigated in Kaposi’s sarcoma (KS), an angiogenic tumour of endothelial origin causally linked to KS herpesvirus (KSHV) infection, and which is the second most common malignancy among AIDS patients (AIDS-associated KS)^18^. In this study, PROX1 was expressed in the large majority (93.3%) of the cases analysed^19^. Interestingly, we and others have demonstrated that infection of LECs with KSHV reduces PROX1 expression^20–22^. Since our previous work showed that the PROX1 downregulation in KSHV-infected LECs reprogrammed the LECs into a more invasive cell type that was dependent on the membrane type 1 matrix metalloproteinase MMP14^20^, we have sought to investigate whether PROX1 regulates the MMP14 levels.

Here we report that PROX1 and MMP14 expressions are inversely correlated and that PROX1 binds and represses transcription from the *MMP14* promoter. Moreover, by manipulating PROX1 expression we could regulate MMP14 expression in an *in vivo* mouse model and change the invasive properties of cancer and blood endothelial cells *in vitro*. Our work reveals that PROX1 regulates MMP14 expression in several cellular contexts, and thus represents an important modulator of normal and cancer cell behaviour.

## Results

### PROX1 and MMP14 expression are inversely correlated

**Figure 1:**
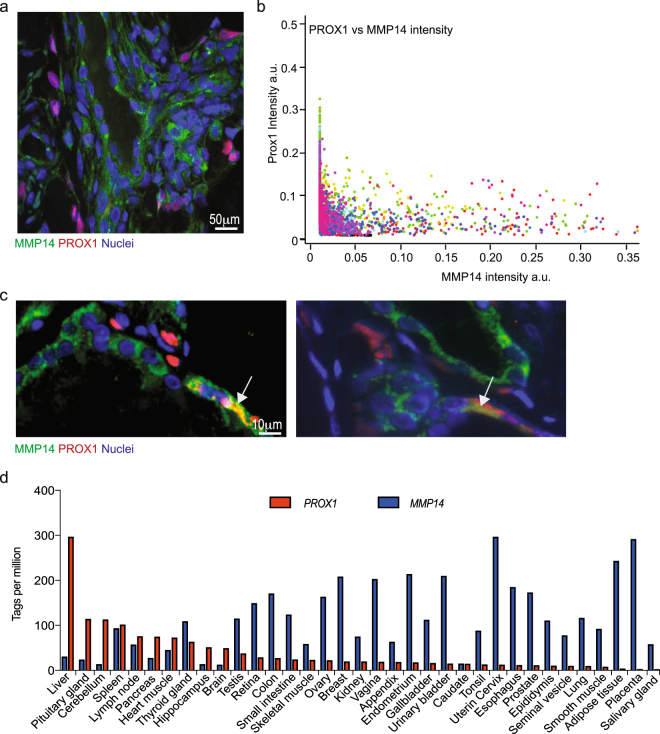
PROX1 and MMP14 expressions are inversely correlated. (**a**) Representative image of a KS section from an AIDS patient stained with MMP14 (green) and Prox1 (magenta) specific antibodies. Nuclei were counterstained with Hoechst 33342. (**b**) MMP14 and Prox1 signal intensities for each cell were quantified from 3 images/section of 10 different patients. Each dot represents a cell and the different colours represent the different patients. (**c**) 57 specimens from TMA were stained as in (**a**), representative images are shown for one benign hyperplasia (left) and one papillary thyroid cancer (right). White arrows indicate cells with Prox1 cytoplasmic staining. (**d**) Transcript levels of Prox1 and MMP14 in different tissues/organs derived from FANTOM5 database. Tags per million for MMP14 (Blue) and Prox1(red) are shown for each tissue/organ. Pearson´s correlation coefficient (r): −0.4515. Significance of the correlation p = 0.0065.

PROX1 is expressed in KS tumours as shown in several publications^19,21–23^. Given our previous observations on MMP14 expression in KS biopsies^20^, we decided to investigate the possible cross-regulation of PROX1 and MMP14 in KS tumours. Sections from ten AIDS-associated KS patients were double-stained for PROX1 and MMP14. The expression of PROX1 and MMP14 appeared inversely correlated throughout the lesions (Fig. 1a,b). In particular, about 98% of the cells with the highest MMP14 intensity had the lowest levels of PROX1, and 95% of the cells presenting the highest PROX1 signal showed low or no MMP14 expression.

A decrease in PROX1 expression was recently observed in thyroid cancer when compared to adjacent healthy tissue, and reintroduction of PROX1 in papillary thyroid cancer (PTC) cell lines abolished the malignant properties (growth, adhesion and invasiveness) of these cells^24^. Therefore, to investigate if reciprocal levels of PROX1 and MMP14 would be observed also in thyroid cancer, we performed double-staining IHC for PROX1 and MMP14 on a tissue microarray (TMA) including 57 specimens of which 44 originated from different thyroid cancers and 13 from benign hyperplasias. We found TMA specimens where only PROX1 (12/57) or MMP14 (14/57) was expressed and samples where both proteins were expressed (22/57) (Fig. 1c). Similar to KS results, in case of PROX1 and MMP14 co-expression in the same tissue section, nuclear PROX1 and cytoplasmic/membrane-associated MMP14 were not found in the same cells, but interestingly, in some specimens cytoplasmic PROX1 and MMP14 were co-localized (Fig. 1c, white arrows). The inversely correlated expression pattern was independent of the stage and the type of the tumour as illustrated in Fig. 1c, where specimens from a benign thyroid hyperplasia and a malignant PTC are shown. Cytoplasmic localization of PROX1 has also been described during lens development^25^ and in thyroid and gastric cancer cells^17,24^. Although PROX1 function in the cytoplasm is not known, it is evident that there it cannot act as a transcriptional repressor.

To assess if the inversely correlated expression of PROX1 and MMP14 occurs also in healthy tissues, we investigated their mRNA levels reported in the FANTOM5 database (www.humanproteinatlas.com). The levels of *MMP14* and *PROX1* were inversely correlated in the majority of the analysed, normal tissues, except in the spleen, where both *PROX1* and *MMP14* mRNA were expressed at intermediate levels (Fig. 1d). Taken together, observations across different cancer types suggest that PROX1 negatively regulates *MMP14* expression.

### PROX1 binds to *MMP14* promoter and represses its transcription

**Figure 2:**
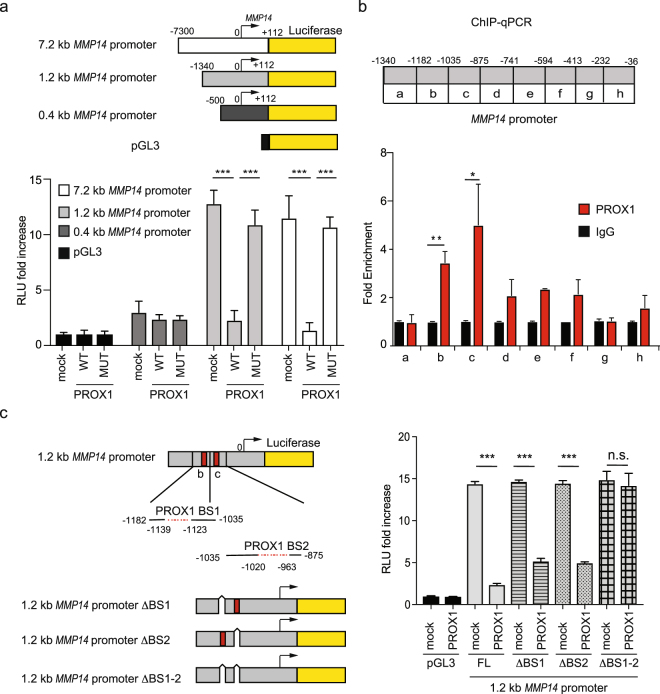
PROX1 binds to the *MMP14* promoter and regulates its expression. (**a**) Upper panel: schematic diagram of the *MMP14* promoter fragments, numbers indicate the bp upstream (−) or downstream (+) of the MMP14 transcription start site (TSS, where bp = 0 is *MMP14* TSS), indicated by the black arrow. Bottom panel: luciferase reporter assay using either the pGL3 backbone or its indicated derivatives depicted above. HeLa cells were co- transfected with the indicated reporter plasmids (0.1 µg) and expression vectors for PROX1 WT or MUT (1 µg) in duplicates. An empty pAMC vector was used as a control (mock). 32 h after transfection, cell extracts were collected and luciferase activities were measured. The graph includes the data from three independent experiments. Error bars represent SD. **(b)** Upper panel: schematic representation of the MMP14 promoter region spanning from bp −1340 to −36. Letters denote the fragments amplified by different sets of primers (a–h) used in the ChIP-qPCR below. Bottom panel: Chromatin immunoprecipitation using either PROX1 or control IgG antibodies followed by qPCR with primers amplifying the indicated regions of the 1.2 kb *MMP14* promoter region. Average fold enrichment over the IgG is shown for three independent experiments; error bars represent SD. (**c**) Left: schematic representation of the 1.2 kb *MMP14* promoter reporter plasmid, the b and c regions immunoprecipitated in (**b**) are marked as red boxes. PROX1 binding site containing regions (BS1 and BS2) are illustrated below as red dotted lines, numbers indicate their position upstream of the *MMP14* TSS. These sequences were deleted in the 1.2 kb *MMP14* promoter reporter plasmid (white boxes), generating the 1.2 kb *MMP14* promoter mutants ΔBS1, ΔBS2, ΔBS1-2. Right: Luciferase assay performed as in (**a**) using 1.2 kb *MMP14* promoter either full length (FL) or the indicated mutants co-transfected with either a pAMC control vector (mock) or a PROX1 WT expression vector. *p < 0.05; **p > 0.01; ***p > 0.001.

To test if PROX1 directly suppresses *MMP14* transcription, we initially performed a luciferase-based reporter assay using plasmids harboring 0.4, 1.2 and 7.2 kb fragments of the 5′-flanking region of the *MMP14* gene upstream of the closest transcription start site (TSS), linked to a firefly luciferase gene (described in^26^ and depicted in the schematic in Fig. 2a, upper panel). The results revealed that Prox1 wild-type (WT) significantly reduced the luciferase activity of the 7.2 kb and of the 1.2 kb *MMP14* promoter fragments (Fig. 2a, lower panel). Notably, a PROX1 mutant (MUT) with point mutations in the Prospero region, responsible in *Drosophila* for the DNA binding and lacking transcriptional activity^27^, had no effect on the *MMP14* reporter activity of any of the constructs tested. Next, we assessed whether PROX1 was negatively regulating *MMP14* promoter activity by direct binding to DNA, as suggested by the lack of effect in the presence of the PROX1 MUT. To this end, we performed ChIP following ectopic expression of PROX1 in iLECs. The samples were then subjected to qPCR using primers recognizing different regions of the *MMP14* promoter (from −1340 to −36 bp upstream of *MMP14* TSS) (diagram in Fig. 2b, upper panel). The ChIP results revealed that PROX1 binds to the *MMP14* promoter in the regions designated as b and c (Fig. 2b) that correspond to sequences previously identified as negative regulatory regions^26^. In silico analysis of these sequences showed that both b and c fragments were harboring putative PROX1-binding sites^28^. The fragment b contains one PROX1-binding site from 11239 to 11223 bp upstream of *MMP14* TSS (PROX1 BS1, Fig. 2c, left panel); whereas the fragment c contains four consecutive PROX1 binding sites from 1020 to 963 bp upstream of *MMP14* TSS (PROX1 BS2, Fig. 2c, left panel). To study the contribution of these putative binding sites to PROX1 transcriptional activity, we generated the ΔBS1 and ΔBS2 mutants, lacking the PROX1 binding sites in the b and c fragment, respectively, as well as ΔBS1-2, devoid of all putative PROX1 binding sites within the b and c fragments of the *MMP14* promoter. The luciferase activity of the ΔBS1 and ΔBS2 was still suppressed by approximately 50% in the presence of WT PROX1 (Fig. 2c, right panel). However, by combining the two deletions (ΔBS1-2) the repression of *MMP14* promoter activity by PROX1 was abolished.

Together, these data indicate that PROX1 is a negative transcriptional regulator of *MMP14* and that the repression occurs through direct PROX1 binding to the *MMP14* promoter regions in the positions from 1139 to 1123 (BS1) and from 1020 to 963 (BS2) bp upstream of *MMP14* TSS.

### PROX1 depletion increases MMP14 levels in murine lymphatic vessels and in human cells

**Figure 3:**
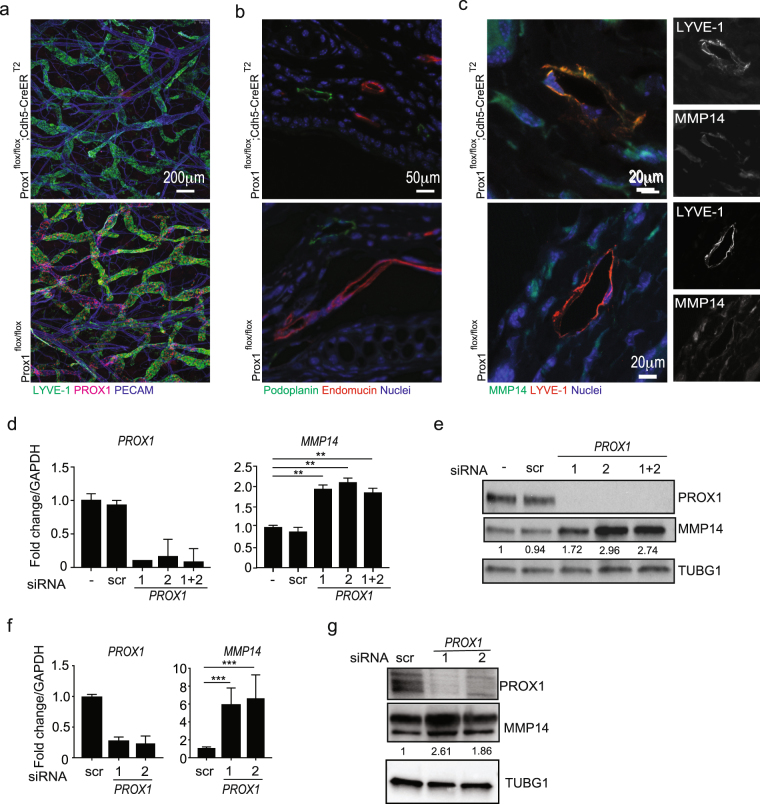
PROX1 depletion increases MMP14 expression *in vivo* and *in vitro*. (**a**) Whole-mount immunofluorescence of 4 weeks old *Prox1*^*flox*^*; Cdh5-CreER*^*T2*^ mice ear skin. Note efficient depletion of PROX1 in the *Prox1*^*flox/flox*^; *Cdh5-CreER*^*T2*^ ear compared to the control. (**b**) Representative images of IHC staining of the ear sections from mice described in (**a**) for podoplanin and endomucin. Nuclei were counterstained with Hoechst 33342. (**c**) Representative images of IHC staining for MMP14 and LYVE −1, a lymphatic endothelial marker, of the ear sections from mice described in (**a**). Nuclei were counterstained with Hoechst 33342. Pearsons’s co-localization index (PC) was calculated using 4 images of three *Prox1*^*flox/flox*^ and four *Prox1*^*flox/flox*^*; Cdh5-CreER*^*T2*^. PC = 0.088 in control mice (N3); PC = 0.640 in Prox1-depleted mice (N = 4); p = 0.01 (t-test). (**d,e**) LECs were transfected with the indicated siRNAs and after 72 h whole cell extracts were analysed by RTqPCR (**d**) for the indicated targets with *GAPDH* as an internal control. Bars represent an average of three independent experiments, error bars show SD across the experiments. (**e**) Immunoblotting for the indicated proteins using γ-tubulin (TUBG1) as a loading control; numbers indicate the intensity of MMP14 band for each sample normalized to the corresponding loading control. Cropped membranes are shown, uncropped membranes can be found in Fig. S3a. (**f,g**) HEK293FT cells were transfected with the indicated siRNAs, 32 h later whole cell extracts were analysed by RTqPCR (**f**) for the indicated targets with *ACT* as an internal control. Bars indicate the average of two independent experiments, error bars show SD; and by immunoblotting (**g**) for the expression of the indicated proteins using γ-tubulin (TUBG1) as a loading control; numbers indicate the intensity of MMP14 band for each sample normalized to the corresponding loading control. Cropped membranes are shown, uncropped membranes can be found in Fig. S3b. **p < 0.01; ***p > 0.001.

Prompted by these observations (Figs 1 and 2) implicating PROX1 as a transcriptional regulator of *MMP14*, we tested if altering the PROX1 levels leads to changes in MMP14 expression *in vivo*. To address this in an *in vivo* physiological setting, we took advantage of the *Prox1*^*flox*^ mouse^29^, crossed with the *Cdh5-CreER*^*T2*^ mouse^30^ to delete *Prox1* specifically in endothelial cells by treatment with 4OH-Tamoxifen (4OHT). The *Prox1*^*flox/flox*^*; Cdh5-CreER*^*T2*^ mice and the Cre-negative *Prox1*^*flox/flox*^ littermate controls were treated with 4OHT at 3 weeks of age. Efficient *Prox1* deletion in dermal lymphatic vessels was confirmed by whole mount immunofluorescence of the ear one week after the treatment, in particular the lymphatic vessels of the *Prox1*^*flox/flox*^*; Cdh5-CreER*^*T2*^ mouse, identified with LYVE-1 staining, were negative for PROX1 (Fig. 3a). Furthermore, we observed no relevant changes in the organization and distribution of PECAM-positive (blood vascular) and LYVE-1 positive (lymphatic) vessels. The other ear was used for paraffin sections, which were stained with endomucin and podoplanin, markers for blood and lymphatic vessels, respectively (Fig. 3b) to further demonstrate that *Prox1* inactivation did not modify the identity or organization of blood and lymphatic vessels in *Prox1*^*flox/flox*^*; Cdh5-CreER*^*T2*^ mice (Fig. 3b). Subsequently, to assess the expression of MMP14 in the PROX1*-*negative lymphatic vessels, mouse ears sections were co-stained with LYVE-1 and MMP14 (Fig. 3c). LYVE-1-positive lymphatic vessels in the *Prox1*^*flox/flox*^*; Cdh5-CreER*^*T2*^ ears showed significantly stronger MMP14 staining when compared to the Cre-negative littermate control ears. The expression of higher levels of MMP14 in the lymphatic vessels of the *Prox1*^*flox/flox*^*; Cdh5-CreER*^*T2*^ ears was also verified by staining for podoplanin, another lymphatic-specific marker (Fig. S1a). Moreover, no change in MMP14 expression in the endomucin-positive blood vessels was observed the in *Prox1*^*flox/flox*^*; Cdh5-CreER*^*T2*^ ears compared to *Prox1*^*flox/flox*^ ears (Fig. S1b). These observations indicate that Prox1 modulates MMP14 levels in the murine LECs *in vivo*.

We next analysed the effect of *PROX1* manipulation on MMP14 expression in human cells. We depleted *PROX1* in human LECs using two different *PROX1*-targeting siRNAs and measured MMP14 expression, comparing it to cells treated with control siRNA (Fig. 3d,e). Upon efficient silencing of *PROX1*, MMP14 mRNA and protein levels were increased about twofold at RNA and about two- to threefold at protein level, thus suggesting that PROX1 functions as a regulator of *MMP14* expression in primary LECs.

PROX1 has been shown previously to regulate LEC fate in part via increased expression of *VEGFR-3* and repression of blood endothelial genes, such as *NRP1*^31^. To study whether upregulation of MMP14 in PROX1-depleted cells coincided with broad LEC fate changes, we analysed *MMP14*, *VEGFR-3* and *NRP2*, a lymphatic-specific marker controlled by the Chicken Ovalbumin Upstream Promoter Transcription Factor 2 (COUP-TF II)^32^, as well as *NRP1* expression in LECs 72 h post siRNA transfection and compared the expression of these genes in LECs and blood endothelial cells, such as BEC and HUVEC. Consistent with previous experiments, *PROX1* depletion induced strong increase in *MMP14* levels. However, *VEGFR-3* and *NRP2* expression levels were not changed and albeit there was a slight increase in *NRP1*, its levels were still significantly lower than in blood endothelial cells (Fig. S1c). Thus, this data supports the notion that PROX1 directly binds to and represses the *MMP14* promoter, and that the induction of MMP14 in *PROX1*-depleted cells precedes broad changes of the LEC fate.

To provide further evidence for the existence of this signaling axis we silenced *PROX1* expression in another PROX1-positive cell type, HEK 293FT (Fig. 3f,g). Similar to LECs, *PROX1* transient silencing increased MMP14 expression in HEK293FT cells both at mRNA and protein levels.

### PROX1 depletion increases *MMP14* levels in cancer cells

**Figure 4:**
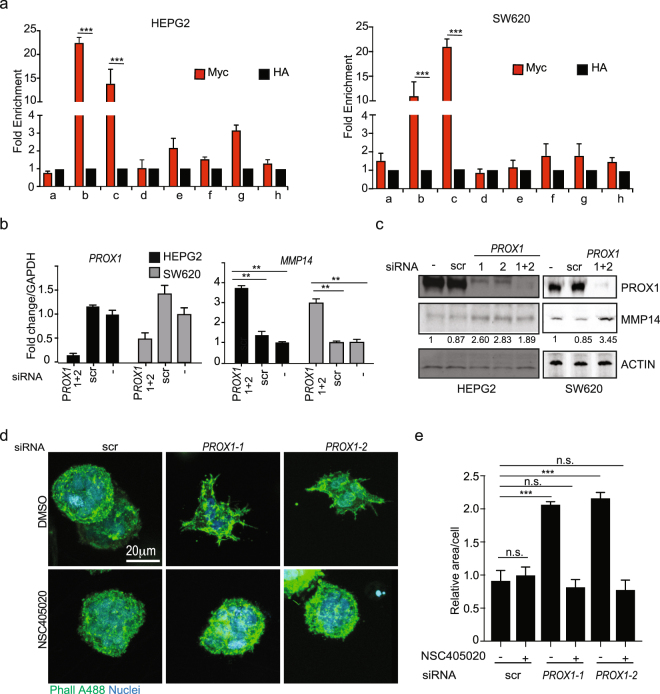
PROX1 silencing increases MMP14 expression and MMP14-dependent 3D sprouting in cancer cells. (**a**) Chromatin immunoprecipitation in HepG2 and SW620 cells ectopically expressing Myg-tagged PROX1. Chromatin was immunoprecipitated using either an anti-Myc antibody or an irrelevant anti-HA antibody and subsequently amplified by qPCR for the indicated regions of the MMP14 promoter (illustrated in Fig. 2b). Average fold enrichment over the anti-HA control is shown for three independent experiments; error bars represent SD. (**b**) HepG2 and SW620 cells were transfected with the indicated siRNAs for 48 h and whole cell extracts were analysed by RTqPCR for the indicated targets with *GAPDH* as an internal control. Bars represent an average of two independent experiments, error bars show SD across the experiments. (**c**) HepG2 and SW620 cells were treated as in (**b**) and whole cell extract was analysed by immunoblot for the indicated proteins using actin as a loading control; numbers indicate the intensity of the MMP14 band normalized to the corresponding loading control. Cropped membranes are shown, uncropped membranes can be found in Fig. S3c,d. (**d**,**e**) 3D fibrin invasion assay with HepG2 cells transfected twice in consecutive days with the indicated siRNAs and embedded in 3D fibrin in the presence of either DMSO or 50 μM of the MMP14 inhibitor NSC405020. After 4 days, fibrin gels were fixed and stained with Phalloidin (Phall A488) and Hoechst 33342. (**d**) Representative images are shown. (**e**) Quantification of three images per condition from two independent experiments (n > 100 cells/condition). Bars represent the average area occupied by each cell cluster and normalized to the scr siRNA and DMSO-treated sample. n.s.: non-significant; **p > 0.01; ***p > 0.001.

We next asked if the PROX1-MMP14 regulation occurs in human cancer cells. Initially, we performed a ChIP-qPCR in HEPG2 hepatocellular carcinoma and SW620 colorectal carcinoma cell lines, transduced with Myc-tagged PROX1 expressing lentivirus. We found that PROX1 bound the same *MMP14* promoter fragments as in iLEC (compare Fig. 2b with Fig. 4a). SW620 and HepG2 endogenously express PROX1 and silencing *PROX1* in these cell lines led to four- (HEPG2) and threefold (SW620) increase of *MMP14* mRNA and a two- to threefold increase of MMP14 protein (Fig. 4b,c).

MMP14 is involved in angiogenic sprouting of endothelial cells^33^ and invasiveness of several solid cancers^34–37^ through its ability to degrade several extracellular matrices^38,39^, including fibrin^40^. We have previously shown that the invasiveness of KSHV-infected LECs into a 3D fibrin matrix is MMP14-dependent^20^. In hepatocellular carcinoma, MMP14 expression has been associated with poor prognosis in HCC patients^41^ and, in HCC cell lines it plays an important role in invasiveness *in vitro* and metastasis *in vivo*^42,43^. Therefore, we next asked whether the observed MMP14 increase associated with *PROX1* depletion would enhance the 3D invasiveness of HepG2 cells. HepG2 cells were transfected with either control siRNA or siRNAs targeting *PROX1* and embedded in 3D fibrin matrix. After 5 days in 3D culture, *PROX1* siRNA-treated cells displayed an increased sprouting when compared to the control siRNA-treated cells, which grew as round spheres (Fig. 4d, upper panels). To study if the observed sprouting was MMP14-dependent, the control and PROX1-depleted cells were treated with the MMP14 specific inhibitor NSC405020 during the 3D fibrin assay. The treatment with MMP14 inhibitor significantly diminished the sprouting of *PROX1* siRNA treated cells, which, similarly to control cells, grew as round spheres in 3D matrix (Fig. 4d, bottom panels and Fig. 4e), thus corroborating the function of MMP14 in the increased invasiveness upon *PROX1* depletion.

Taken together, these results indicate that PROX1 binds to the *MMP14* promoter and thereby negatively regulates its expression in human tumour cells. Moreover, *PROX1* depletion enhances HEPG2 MMP14-dependent invasiveness in 3D fibrin matrix.

### Reintroduction of PROX1 inhibits 3D sprouting and invasiveness of endothelial and cancer cells

**Figure 5:**
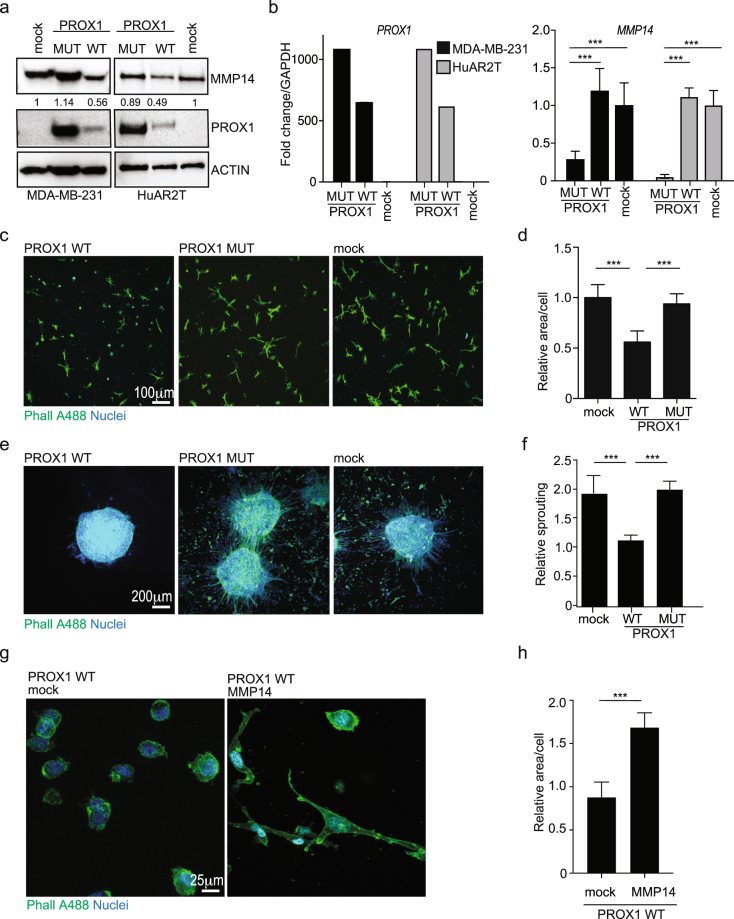
PROX1 reintroduction decreases MMP14 expression and reduces 3D invasiveness in BEC and cancer cells. (**a**) MDA-MB-231 and HuAR2T were treated with the indicated siRNAs for 72 h, and whole cell extracts were analysed by immunoblotting for the indicated proteins using actin as a loading control; numbers indicate the intensity of MMP14 band for each sample normalized to the corresponding loading control. Cropped membranes are shown, uncropped membranes can be found in Fig. S3e,f. (**b**) The samples in (**a**) were analysed by RTqPCR for the indicated targets with GAPDH as an internal control. Bars represent the average of three independent experiments, error bars show SD. (**c**) 3D fibrin invasion assay with MDA-MB-231 transduced with lentiviruses expressing PROX1 WT or MUT and stained with Phalloidin (Phall A488) and Hoechst. Empty vector was used as a control (mock). Representative images are shown. (**d**) Quantification of three images per condition described in (**c**) from three independent experiments (n > 200 cells/condition). Bars represent the average area occupied by each cell and normalized to the mock infected cells. **(e)** 3D sprouting assay of HuAR2T spheroids, treated and stained as in (**c**). Representative images are shown. **(f)** Quantification of three independent experiments described in (**e**). Bars represent the average of the total area of each spheroid divided by the area of the non-invading cells. Three spheroids per condition were quantified in three independent experiments. Error bars indicate SD. **(g,h)** MDA-MB-231 cells were transduced with PROX1 WT-expressing lentivirus for 24 h and subsequently transfected with a control vector (mock) or MMP14 expression vector for 24 h. Cells were then embedded in fibrin and treated as in (**c**). **(g)** Representative enlarged images are shown. **(h)** Quantification of three images/condition for two independent experiments (n > 100). Bars represent the average of the total area occupied by each cell and normalized to the mock transfected cells. Error bars indicate SD. **p < 0.005; ***p > 0.001.

Since silencing of *PROX1* increased MMP14 levels, the reintroduction of PROX1 into PROX1-negative cells should decrease MMP14 expression. To test this, we chose HuAR2T, an HuVEC cell line, and MDA-MB-321, an invasive breast cancer cell line, both expressing high levels of MMP14, but no PROX1. We transduced both cell lines with a lentivirus encoding either WT or DNA binding-deficient PROX1. *MMP14* mRNA decreased dramatically upon ectopic expression of PROX1 WT, but not PROX1 MUT. The decrease in MMP14 protein upon PROX1 WT expression was 50% less compared to the control (Fig. 5a,b).

MMP14 plays a pivotal role in BEC sprouting as well as in solid cancer invasiveness^33,34,44^. In particular in breast cancer, MMP14 expression is a marker of increased cell invasiveness^35,45^. Therefore, we tested if the PROX1-induced decrease in MMP14 affects the ability of endothelial and cancer cells to invade into 3D crosslinked fibrin. First, we confirmed that *MMP14* silencing decreased the 3D sprouting growth of MDA-MB-231 cells in 3D fibrin (Fig. S2a). Next, MDA-MB-231 cells ectopically expressing PROX1 WT or MUT, as well as control vector-transduced cells were embedded in fibrin for 3 days. As shown in Fig. 5c,d the PROX1 WT expressing cells had a significantly decreased ability to invade into the matrix as compared to the PROX1 MUT or mock-treated cells.

MMP14 inhibition by the NSC405020 treatment reduced the 3D invasive sprouting of HuAR2T (Fig. S1b), demonstrating that the invasive sprouting of these cells is MMP14 dependent. Moreover, HuAR2T spheroids expressing ectopic PROX1 WT sprouted significantly less than the PROX1 MUT-expressing or untreated spheroids (Fig. 5e,f).

To confirm that the PROX1 expressing cells were less invasive in 3D fibrin, both HuAR2T spheroids and MDA-MB-231 single cells ectopically expressing PROX1 WT were stained with antibody specific for PROX1 and the invasiveness of the PROX1 positive cells was compared to that of PROX1 negative cells within the same fibrin gel. Results shown in Fig. S2c–f illustrate that the PROX1-expressing cells sprouted/invaded significantly less than the PROX1-negative cells. To test if the invasive phenotype of PROX1 WT expressing MDA-MB-231 cells could be rescued by MMP14, we reintroduced MMP14 by transient transfection. As shown in Fig. 5g,h the inhibition of invasiveness by PROX1 WT was restored by reintroduction of MMP14.

In conclusion, these results show that PROX1 acts as a negative regulator of *MMP14* expression and MMP14-dependent 3D invasion in multiple cells types, such as BEC and breast cancer cells.

## Discussion

MMP14 expression is controlled at different levels, and its promoter differs from those of other MMPs^46^. The transcription factors Sp1^26^, E2F^47^, Egr1^48^ and HIF2^49^ have been identified as *MMP14* transcriptional activators, and to our knowledge, no repressors of *MMP14* have been found so far. Here we show that PROX1 suppresses *MMP14* transcription. This discovery stems from our previous observation that KSHV-infection of LECs leads to a decrease in *PROX1* expression with a concomitant increase in MMP14 expression and MMP14-dependent invasiveness^20^.

Our data further demonstrates increased levels of MMP14 in *Prox1-*depleted mouse dermal lymphatics suggesting that PROX1 suppresses *MMP14* expression under physiological conditions. In the lymphatic vasculature, the role of MMP14 is controversial. In corneal lymphangiogenesis MMP14 degrades ECM components and activates MMP2 to promote migration and sprouting of the LECs^50^, and in LEC spheroids it stimulates cell sprouting in a 3D crosslinked matrix^20^. However, recent data shows that *MMP14* depletion in LECs increased corneal lymphangiogenesis thus implicating MMP14 as an inhibitor of this process^51^. The physiological levels of PROX1 in LECs could thus contribute to maintain MMP14 expression at appropriate levels during lymphangiogenesis. This is supported by the phenotype of the *Foxc2*-deficient mice that fail to downregulate *Prox1* in the developing lymphatic vessels. These *Foxc2-*deficient vessels display defective capillary sprouting and remain in an immature-capillary-like state^52^, which could be influenced by the lack of MMP14 expression due to the aberrant *Prox1* expression. On the other hand, haploinsufficiency of PROX1 has also been reported to promote abnormal lymph leakage^53^, which could be due to untimely MMP14 expression. However, whether these phenotypes, linked to an increase or decrease in PROX1 expression, are induced by aberrant levels of MMP14, remains to be confirmed.

Activation of the TWIST-AKT2 axis and increased metastatic invasiveness have been linked to PROX1 downregulation in HCC^16^. As MMP14 has been also shown to enhance HCC metastasis^42^, it is possible that MMP14 upregulation and the concomitant increase in the MMP14-dependent invasive sprouting in HepG2 observed here in response to PROX1 downregulation, could contribute to the malignant phenotype of HCC.

A recent work reported *PROX1* mRNA downregulation and the unexpected cytoplasmic mis-localization of the PROX1 in PTC^24^. In that study, reintroduction of nuclear PROX1 into a PTC cell line BCPAP suppressed the invasive phenotype of the cells implying that PROX1 inactivation contributes to thyroid carcinoma invasiveness. This, together with our data on the inversely correlated expression of PROX1 and MMP14 in the thyroid cancer biopsies, suggests that the increased invasiveness could be due to an increase in MMP14 expression.

Therefore, by governing the expression of *MMP14*, PROX1 might function as a gate-keeper that ensures appropriate MMP14 levels. This is also supported by the observation that PROX1 is expressed in mammary stem cells^54^, but not in breast cancer where PROX1 gene is silenced through hyper-methylation^55^ and MMP14 is highly expressed. The epigenetic silencing of PROX1 might be necessary for the MMP14-dependent invasiveness since here we show that PROX1 reintroduction in MDA-MB-231 significantly reduces MMP14 expression and 3D-invasiveness.

Taken together, demonstration that PROX1 negatively regulates *MMP14* in normal tissues and in cancer supports the general nature of this signalling axis and its potential importance in physiological and pathological processes.

## Material and Methods

### Cell culture

HeLa (ATTC:CCL-2™) and HEK293FT (Thermofisher Scientific, R70007) cells were grown in Dulbecco modified Eagle’s medium (DMEM) supplemented with 10% Foetal Calf serum (FCS) and 1% L-Glutamine. HepG2 (ATTC:HB-8065), SW620 (ATCC:CCL-227™) and MDA-MB-321 (ATCC:CRM-HTB-26™) were cultured in RPMI 1640 supplemented with 20% FCS and 1% L-Glutamine. HuAR2T, human umbilical vein endothelial cells (HuVEC), conditionally immortalized with a doxycycline-inducible human telomerase reverse transcriptase (hTERT) and Simian virus 40 (SV40) large T antigen transgene expression, were grown in EGM2-MV medium supplemented with 200 ng/ml of Doxycycline. Human dermal LECs, HuVEC, BEC (purchased from Lonza), and iLECs, the intestine-derived LECs immortalised with the HPV E6 and E7 proteins^56^, were grown in EGM2-MV (Lonza). All cells were propagated at standard conditions (37 °C, 5% CO_2_).

### Antibodies, plasmids, siRNAs, inhibitors

The following antibodies were used for immunoblots: mouse anti-actin (Santa Cruz Biotechnology; SC-8432), mouse anti-TBG1 (Sigma-Aldrich; T6557), goat anti-hPROX1 (R&D systems; AF2727), rabbit anti-MMP14 (R&D Systems; EP1264Y). Secondary antibodies HRP-conjugated were from Chemicon (Millipore) and Cell Signaling Technology.

For the staining of mouse ears the following antibodies were used: rat anti-mouse PECAM1 (BD-Biosciences; 553370), rabbit anti-mouse LYVE-1 (103-PA50AG, Reliatech), goat anti-PROX1 (R&D AF2727), rat anti mouse endomucin (Santa Cruz sc-65495), hamster anti mouse podoplanin (Developmental Studies hybridoma bank 8.1.1) and mouse monoclonal anti-MMP14 (LEM clone; Millipore). Secondary antibodies conjugated to Cy3, Alexa Fluor 488 or 647 were obtained from Jackson ImmunoResearch or Life Technologies (Thermofisher).

Reporter plasmids harbouring the MMP14 promoter fragments upstream of the firefly luciferase gene were generated using pGL3 backbone^26^. MMP14 promoter plasmids lacking the PROX1 binding sites (indicated as ΔBS1 and ΔBS2) were generated from the pGL3-1.2 kb MMP14 promoter using NEBuilder HiFi assembly kit. The backbone and the insert were amplified by PCR using the following primers (forward; reverse):

ΔBS1: TTGGATCTTAGATTTCGCTGATAGTCTAGTTTTC; GTGAGAGAGGACAGAGGTAGTGC.

ΔBS2: TTTTGTTCTGAGTCCAGTAAGTCCCTAAAG; TTTTGTTCTGAGTCCAGTAAGTCCCTAAAG.

and ligated using HiFi Assemby Master Mix (NEB) using the following ligation oligonucleotides:

ΔBS1: CTACCTCTGTCCTCTCTCACTTGGATCTTAGATTTCGCTG.

ΔBS2: CCAGACCTGTCTAGTTCCCATTTTGTTCTGAGTCCAGTAAG.

*PROX1* gene was cloned in a pAMC plasmid and the mutation N624A and N626A in the *PROX1* mutant (MUT) was introduced by site-directed mutagenesis^8,27^. Subsequently the *PROX1* wild-type (WT) and *PROX1* MUT were cloned into the pSIN lentiviral vector^57^. The inserts were verified by sequencing.

The pSport6-MMP14 expression plasmid, containing the *MMP14* gene transcribed from CMV-IE promoter, was obtained from Genome-Biology Unit, University of Helsinki.

Two different Stealth RNAi™ targeting *PROX1* (HSS 108596-7) and siRNA Negative Control (12935200) were purchased from Invitrogen. *MMP14*-targeting siRNAs (SI03648841; SI00071176) and the corresponding control siRNA (1027281) were purchased from Qiagen. The MMP14 inhibitor NSC405020 was obtained from Sigma-Aldrich (2044451).

### Transfection, lentivirus production and transduction

DNA and RNA transfections were performed using Lipofectamine 2000 and Lipofectamine RNAiMAX, respectively according to the instructions provided by the manufacturer. Lentiviruses were produced in HEK293FT transfected with pLP1, pLP2, pVSVg and either an empty pSIN or pSIN-*PROX1* WT or pSIN-*PROX1* MUT. After 72 h, lentivirus particles were precipitated with PEG-IT (System Biosciences) according to the vendor’s instructions. Transduction of cells with lentiviruses was done by spinoculation (450 g, 30 min, RT) in the presence of 8 μg/ul of polybrene^20^.

### RTqPCR

Total RNA was extracted from cells using Nucleospin Spin RNA extraction kit (Macherey Nagel) and reverse transcribed as described in^20^. Transcript levels were measured in triplicate using unlabelled primers and SYBR GREEN reaction mix (Fermentas). The following primer sequences were used (forward; reverse):

*PROX1*: TGTTCACCAGCACACCCGCC; TCCTTCCTGCATTGCACTTCCCG.

*MMP14*: GCAGAAGTTTTACGGCTTGCAA; CCTTCGAACATTGGCCTTGAT.

*NRP1*: GCATGAAGGCAGACAGAGATG; CTGTCGGCCATACTCATTGAA.

*NRP2*: GGGAACACCCAAGACAGTGA; TCAAACCTTCGGATGTCAGG.

*VEGFR3*: GACAGCTACAAATACGAGCATCTG; CTGTCTTGCAGTCGAGCAGA.

*GAPDH*: TCAACGACCCCTTCATTGAC; ATGCAGGGATGATGTTCTGG

*Actin*: TCACCCACACTGTGCCATCTACGA; CAGCGGAACCGCTCATTGCCAATGG.

Data were normalized to the cellular housekeeping genes *GAPDH* or *ACT*. Experiments were performed two or three times in three technical replicates, the graphs represent an average of the fold change and error bars indicate SD ± 95%CI across the different experiments.

### Western blotting

Cells were lysed in RIPA buffer (150mM-NaCl; 1%-Igepal CA630-0.5% Na-deoxycholate-0.1% SDS-50mM; Tris-HCl-PH 8.0) supplemented with phosphatase (Pierce™ 88667) and protease (Pierce™ 88666) inhibitors. Pre-cleared cell extract was mixed with 5XLaemmli buffer and loaded on Criterion TGX precast gels (Bio-Rad). Gels were run for 40 min at 55 mA and transferred on nitrocellulose membranes using trans-blot Turbo Transfer system (Bio-Rad). Membranes were blocked in 2.5% non-fat dry milk in TBS-T (0, 1%Tween) for one hour at RT, gently rocking, and incubated O/N at 4 °C with the primary antibody diluted in blocking solution, membranes were then incubated with the appropriate HRP-conjugated secondary antibody (Chemicon International) for 1 h at RT. Luminescent signal was revealed using WesternBright Sirius detection kit (Advansta). Experiments were repeated at least two times.

Where indicated, band intensities were quantified using the Fiji software (https://imagej.net/Fiji). For each sample, MMP14 band intensity was normalized to the corresponding loading control, relative band intensities are shown.

### Luciferase-based reporter assay

5000 HeLa cells/well were seeded in a 96-well plate. Next day cells were transfected in the presence of Lipofectamine2000 with 25 ng of the Renilla reporter, 50 ng of the indicated reporter plasmid, 1000 ng of either pAMC-*PROX1* (WT or MUT) or 1000 ng of the corresponding control vector. 36 hours after transfection cells were lysed in 30 µl/well of 1X reporter lysis buffer (Promega). Luciferase activity was measured using a luciferase buffer according to the manufacturer’s instructions (Promega). The assay was repeated two or three times using two biological replicates and the error bars represent SD ± 95%CI across all experiments. For each sample, the firefly luciferase values were normalized to the Renilla luciferase values.

### Chromatin immunoprecipitation (ChIP)

iLECs^52,57^ were transduced with *PROX1*-pSD44^12^ and 72 hrs later processed according to simpleChIP Kit (Cell Signalling Technology) instructions. Chromatin was precipitated using Goat anti-human PROX1 antibody (R&D Systems, AF2727) or normal goat IgG antibody (Santa Cruz Biotechnology, sc-2028).

HepG2 and SW620 were transduced with a pSIN-*PROX1* WT encoding lentivirus, chromatin was precipitated with mouse monoclonal anti Myc-tag antibody (Cell Signaling Technology #2276) or a control HA-tag mouse monoclonal antibody (HA.11; MMs-101R-200, Biolegend).

After DNA purification (Macherey-Nagel PCR-purification Kit, 740609), the following primers were used to amplify different domains of the *MMP14* promoter (forward; reverse):

a: AGGACCTGAAAAGCTTTTCAT; GGGTGGACAGAAATTAGGT

b: TCCTCTATTCCTTCCTTTGCT; AAGCACAACAGAAGCAGG

c: CTTTTCCAGACCTGTCTAGTTC; CACTGAAAAAGGAGGCAATTC

d: TAGAGGTGGAACTAAACCCC; CCTTTAATTGGAACTCGCTGG

e: TGCAGCCACATTACAAATGA; TGTCTATGTCCCTCCCTCTG

f: CATAGACAGTTGTCTACAGGG; TATTGGGGGCTATGTGGCTA

g: CCACATAGCCCCCAATAATT; TTGTGGTGCAGGCTGCCATC

h: AAGTCTCCCACATCCCGTCC; CCAGTGCCCTCCTTTCCTGGTTG

### 3D sprouting and invasion assays

4000 HuAR2T cells were seeded in the 0.5% low melting point agarose-coated round bottom 96-well plate. Spheroids were allowed to form for 36–48 hours, harvested and embedded in 3 mg/ml plasminogen-free fibrinogen (Calbiochem)^20^. Spheroids were grown in fibrin for 4 days. Alternatively, a single-cell suspension of 5000 MDA-MB-321 cells were embedded in fibrinogen and followed for 72 h. The fibrin droplets were fixed in 4% PFA and stained with Alexa488-conjugated Phalloidin (Life Technologies), nuclei were counterstained with Hoechst 33342 (1ug/ml). Images were taken using Zeiss LSM-780 as previously reported^20^.

### Mice

*Cdh5-CreER*^*T2*^ and *Prox1*^*flox*^ lines were described previously^29,30^. For induction of Cre recombination, 1 mg of 4-hydroxytamoxifen (4-OHT), dissolved in peanut oil (10 mg/ml), was administered to 3 weeks old mice by intraperitoneal injection. Mice were analysed 1 week after treatment.

Experimental procedures were approved by the Uppsala Laboratory Animal Ethical Committee (permit number:C130/15) and performed in accordance with relevant guidelines and regulations.

### Tumour tissues

Paraffin embedded KS sections were kindly obtained from Dr. Justin Weir (Charing Cross Hospital, and the London Clinic, London), The research was covered by Riverside Research Ethics Committee (Study title: Kaposi’s Sarcoma Herpes Virus Infection And Immunity, REC reference: 04/Q0401/80); all patients gave written informed consent.

Tissue microarray of thyroid cancers was constructed from surgical specimens obtained from the archives of the Department of Pathology, Helsinki University Hospital, according to Finnish laws and regulations by permission of the director of the health care unit. The tissue samples were de-identified and analysed anonymously.

### Immunohistochemistry

Tissue sections were deparaffinized and rehydrated. Antigen retrieval was performed by heating in 0.1 M citrate buffer (pH 6.0) prior to blocking with 5% normal donkey serum for 1 hour at RT. Sections were stained with the indicated primary antibody O/N at 4 °C in humidified chambers and with the appropriate secondary antibody for 1 hour at RT, nuclei were counterstained with Hoechst 33342 (1ug/ml).

For the whole mount immunofluorescence, the dorsal side of the ear was fixed in 4% paraformaldehyde (PFA), permeabilized in 0.3% Triton X-100 in PBS (PBST), blocked in PBST with 3% milk. Primary antibodies were incubated at 4 °C overnight in blocking buffer. After washing, the samples were incubated with fluorescence-conjugated secondary antibodies before further washing and mounting in Mowiol.

Samples were scanned in a Panoramic 250 viewer (Genome Biology Unit, Research Programs Unit, University of Helsinki). Alternatively, confocal Z-stack images were acquired as previously described^20^.

### Analysis of gene expression database

To assess the reciprocal expression levels of *PROX1* and *MMP14* the FANTOM5 (http://www.proteinatlas.org/) was analysed. The data were obtained by CAP analysis of gene expression (CAGE) and reported by Tags per million.

### Statistical analysis

Student T-test was used to assess whether the difference between the experimental groups was statistically significant. Analyses were performed using GraphPad PRISM6 and 7. Co-localization of proteins in the mouse ears was quantified using JACoP plug-in in the Fiji software (https://imagej.net/Fiji). KS sections were analysed using the ANIMA software^58^.

The sprouting of single invasive cells in fibrin was quantified with the Cell Profiler pipeline (http://cellprofiler.org/). For each image stack the area of each cell (visualized as Phalloidin-stained area related to one nucleus) was measured. Each stack included approximately 20–40 cells, and for each independent experiment three random stacks were quantified. The invasion of spheroids was quantified from the Phalloidin A488 labelled image by calculating the total area occupied by the spheroid and the area occupied by the central non-invasive area using the Image J software. The average ratio between the total area of the spheroids and the non-invasive body of the spheroid is indicated in the graphs.

### Data availability

The gene expression data in Fig. 1d are freely available at http://www.proteinatlas.org. The data generated or analysed during this study are included in this published article (and its Supplementary Information files).

## Electronic supplementary material

Supplementary Figures S1-S2-S3
