# Supplementary material for: PROX1 is a transcriptional regulator of MMP14
Source: Sci Rep. 2018 Jun 22;8:9531. doi: 10.1038/s41598-018-27739-w (PMC6015061; doi:10.1038/s41598-018-27739-w)
Supplement: Supplementary file 1 — Supplementary Figures S1-S2-S3 [file 41598_2018_27739_MOESM1_ESM.pdf]

PROX1 is a transcriptional regulator of MMP14

Silvia Gramolelli, Jianpin Cheng, Ines Martinez-Corral, Markus Vähä-Koskela, Endrit Elbasani, Elisa Kaivanto, Ville Rantanen, Krista Tuohinto, Sampsa Hautaniemi, Mark Bower, Caj Haglund, Kari Alitalo, Taija Mäkinen, Tatiana V. Petrova, Kaisa Lehti and Päivi M. Ojala*

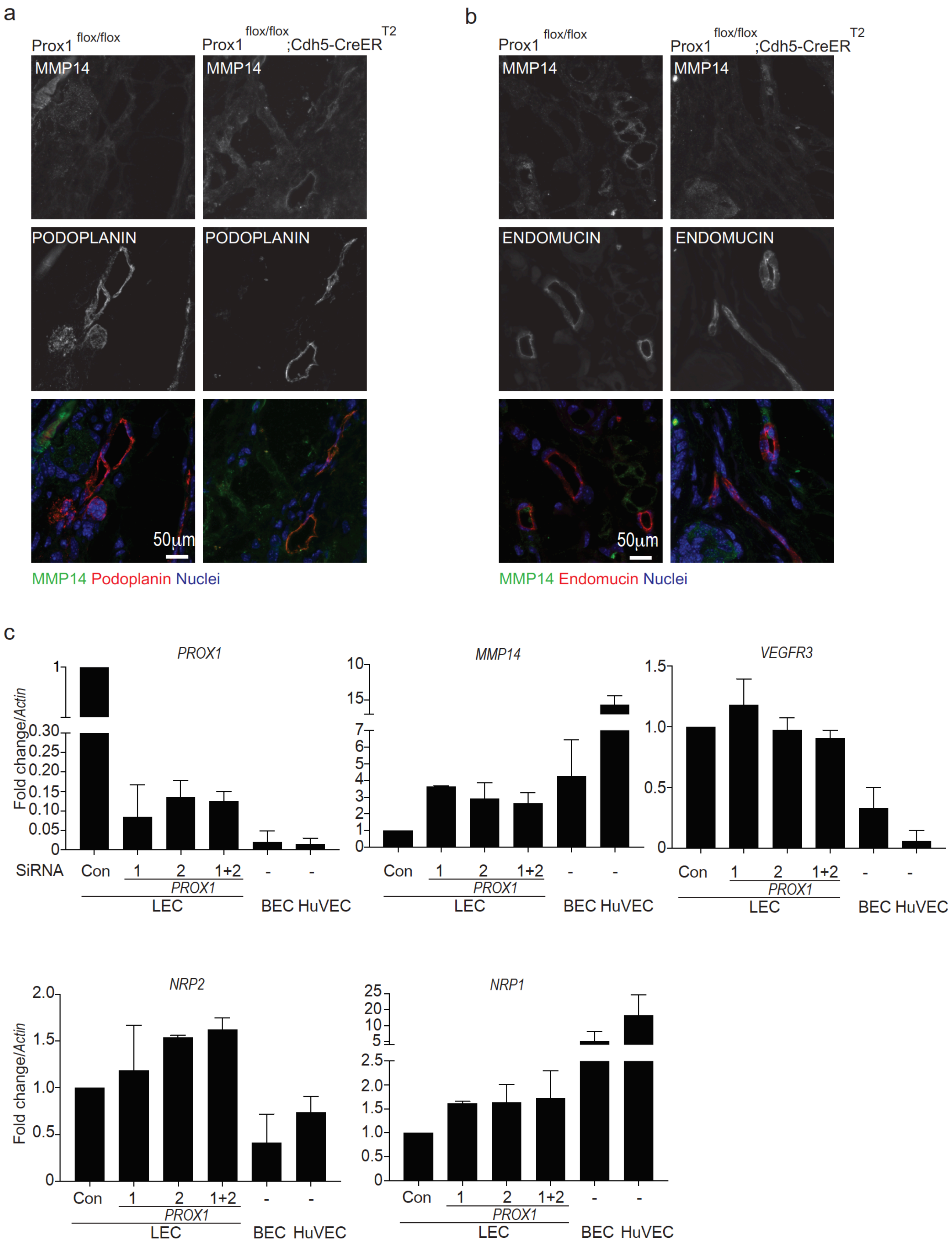
a
Prox1 flox/flox
Prox1 flox/flox;Cdh5-CreER T2
MMP14
PODOPLANIN
50μm
MMP14 Podoplanin Nuclei
b
Prox1 flox/flox
Prox1 flox/flox;Cdh5-CreER T2
MMP14
ENDOMUCIN
50μm
MMP14 Endomucin Nuclei
c
PROX1
MMP14
VEGFR3
NRP2
NRP1
Fold change/Actin
SiRNA
Con
1
2
1+2
-
PROX1
LEC
BEC HuVEC

Figure S1. PROX1 depletion does not affect the expression of other LEC and BEC markers. **(a,b)** Representative images of the ear sections from mice described in (Figure 3a-c) and stained by specific antibodies against MMP14 and podoplanin (a), or MMP14 and endomucin (b). Nuclei were counterstained with Hoechst 33342. **(c)** LECs transfected with the indicated siRNAs for 72h were analysed by RTqPCR for *PROX1 MMP14*, *VEGFR3, NRP2* and *NRP1* expression and their expression was compared to untreated BECs and HuVECs. *ACT* was used as an internal control. Bars represent an average of two independent experiments, error bars show SD across the experiments.

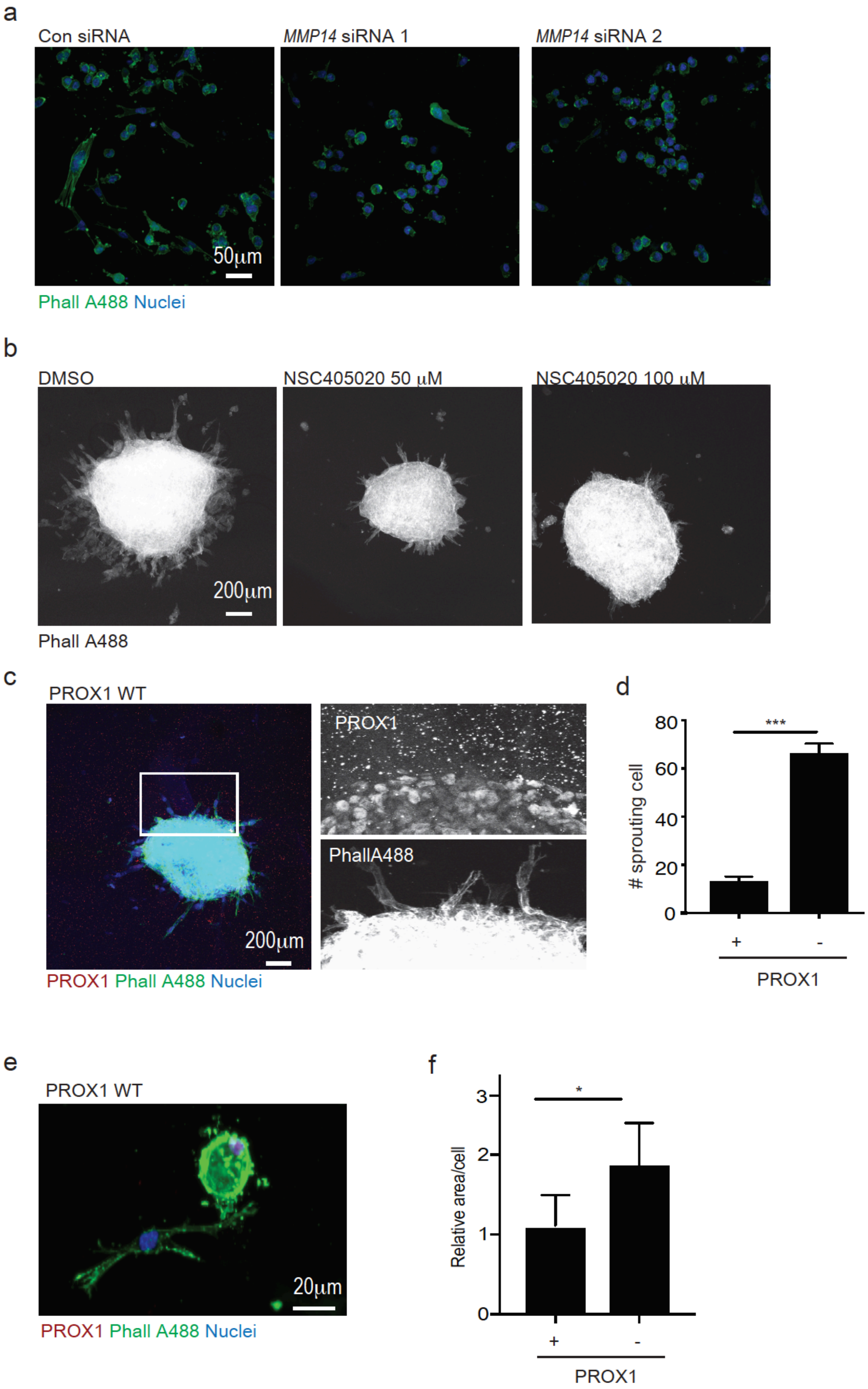
a
Con siRNA
MMP14 siRNA 1
MMP14 siRNA 2
50μm
Phall A488 Nuclei
b
DMSO
NSC405020 50 μM
NSC405020 100 μM
200μm
Phall A488
c
PROX1 WT
PROX1
PhallA488
200μm
PROX1 Phall A488 Nuclei
d
sprouting cell
80
60
40
20
0
***
+
-
PROX1
e
PROX1 WT
20μm
PROX1 Phall A488 Nuclei
f
Relative area/cell
3
2
1
0
*
+
-
PROX1

Figure S2. Ectopic expression of PROX1 in BEC and breast cancer cells impairs their sprouting in 3D fibrin. (a) MDA-MB-231 cells were treated with the indicated siRNAs and 24h later embedded in 3D fibrin for 4 days. Fibrin gels were stained with Phalloidin (Phall A488) and nuclei counterstained with Hoechst 33342. Representative images are shown. **(b)** HuAR2T spheroids embedded in 3D fibrin were treated with DMSO or the indicated concentrations of the MMP14 inhibitor NSC405020 and grown for 4 days. Fibrin gels were stained as in (a). Representative images are shown. **(c,d)** HuAR2T spheroids ectopically expressing PROX1 WT were embedded in 3D fibrin for 4 days and stained with PROX1 specific antibody and Phall A488. Nuclei were counterstained with Hoechst 33342. **(c)** left panel: representative image is shown, and the area enclosed in the white square is enlarged to show PROX1 (top right panel) and phalloidin (bottom right panel) in separate channels. **(d)** Quantification of the number of PROX1 positive (+) and negative (-) cells sprouting from the spheroid main body. Three spheroids from two independent experiments were quantified (n=6). Bars show the average, and error bars the SD**. (e,f)** MDA-MB-231 cells ectopically expressing PROX1 WT were subjected to the fibrin assay and stained as in (c). **(e)** Representative image is shown. **(f)** Quantification for PROX1 positive (+) or negative (-) cells in three images/condition for two independent experiments (n>100). Bars represent the average of the total area occupied by PROX1 positive or negative cells and normalized to the area of PROX1 positive cells. Error bars indicate SD.*: p<0.005; ***: p>0.001.

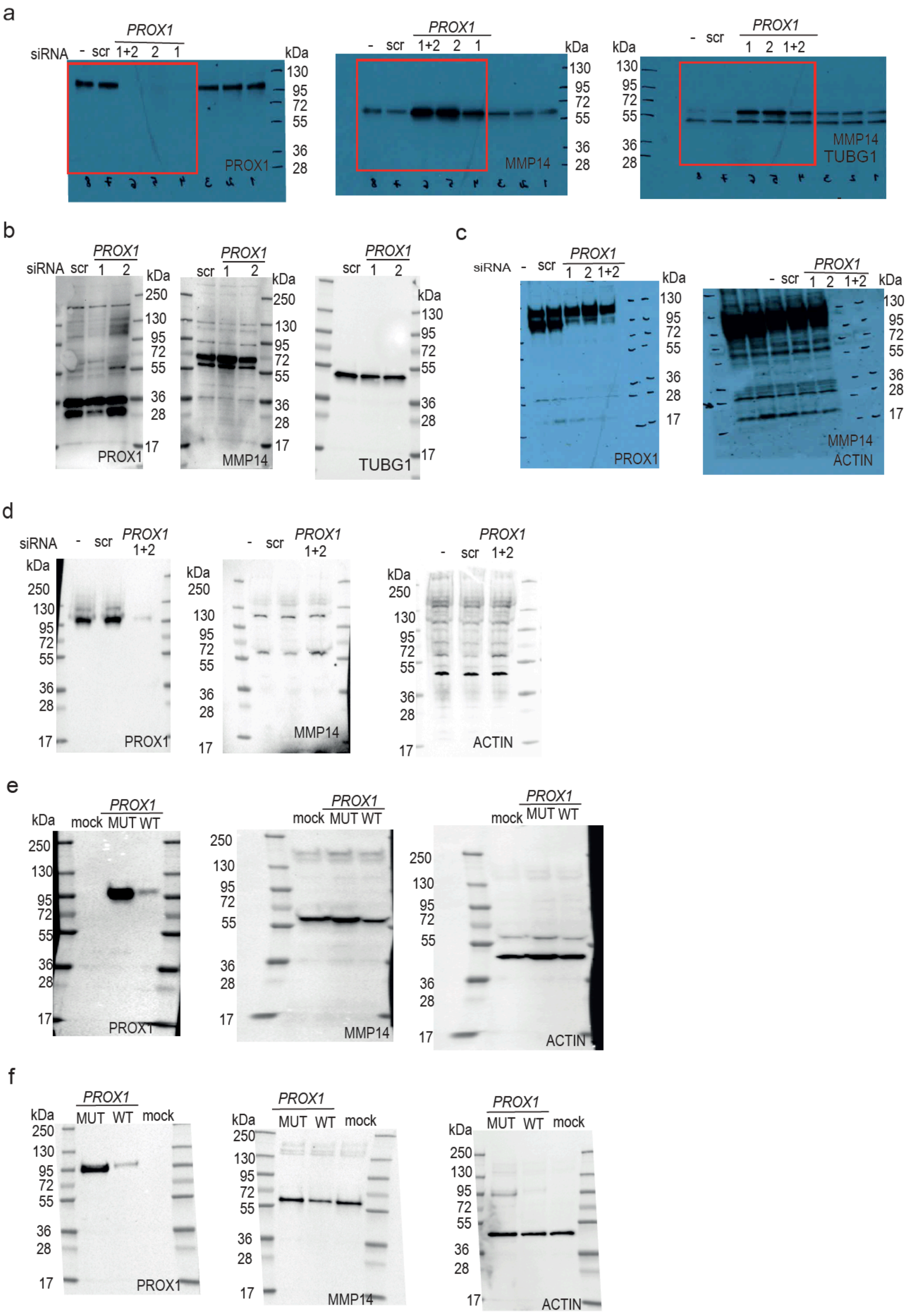
a
siRNA - scr PROX1 1+2 2 1
kDa 130 95 72 55 36 28
PROX1
- scr PROX1 1+2 2 1
kDa 130 95 72 55 36 28
MMP14
kDa 130 95 72 55 36 28
- scr PROX1 1 2 1+2
MMP14
TUBG1
b
siRNA scr PROX1 1 2
kDa 250 130 95 72 55 36 28 17
PROX1
scr PROX1 1 2
kDa 250 130 95 72 55 36 28 17
MMP14
scr PROX1 1 2
kDa 130 95 72 55 36 28 17
TUBG1
c
siRNA - scr PROX1 1 2 1+2
kDa 130 95 72 55 36 28 17
PROX1
- scr PROX1 1 2 1+2
kDa 130 95 72 55 36 28 17
MMP14
ACTIN
d
siRNA - scr PROX1 1+2
kDa 250 130 95 72 55 36 28 17
PROX1
- scr PROX1 1+2
kDa 250 130 95 72 55 36 28 17
MMP14
- scr PROX1 1+2
kDa 250 130 95 72 55 36 28 17
ACTIN
e
kDa mock PROX1 MUT WT
250 130 95 72 55 36 28 17
PROX1
mock PROX1 MUT WT
250 130 95 72 55 36 28 17
MMP14
mock PROX1 MUT WT
250 130 95 72 55 36 28 17
ACTIN
f
kDa PROX1 MUT WT mock
250 130 95 72 55 36 28 17
PROX1
kDa PROX1 MUT WT mock
250 130 95 72 55 36 28 17
MMP14
kDa PROX1 MUT WT mock
250 130 95 72 55 36 28 17
ACTIN

Figure S3. **Uncropped western blot membranes.** (a-f) Uncropped western blot membranes of the experiments presented in the manuscript. The red squares highlight the portion shown in the main figures. The detected proteins are indicated at the right bottom corner of each membrane; molecular weight is shown on the side of each membrane.
